# Social familiarity modulates group living and foraging behaviour of juvenile predatory mites

**DOI:** 10.1007/s00114-012-0903-7

**Published:** 2012-03-15

**Authors:** Markus A. Strodl, Peter Schausberger

**Affiliations:** Group of Arthropod Ecology and Behavior, Division of Plant Protection, Department of Crop Sciences, University of Natural Resources and Life Sciences, Peter Jordan-Strasse 82, 1190 Vienna, Austria

**Keywords:** Familiarity, Limited attention, Association behaviour, Life history, Foraging, Phytoseiidae

## Abstract

Environmental stressors during early life may have persistent consequences for phenotypic development and fitness. In group-living species, an important stressor during juvenile development is the presence and familiarity status of conspecific individuals. To alleviate intraspecific conflicts during juvenile development, many animals evolved the ability to discriminate familiar and unfamiliar individuals based on prior association and use this ability to preferentially associate with familiar individuals. Assuming that familiar neighbours require less attention than unfamiliar ones, as predicted by limited attention theory, assorting with familiar individuals should increase the efficiency in other tasks. We assessed the influence of social familiarity on within-group association behaviour, development and foraging of juvenile life stages of the group-living, plant-inhabiting predatory mite *Phytoseiulus persimilis*. The observed groups consisted either of mixed-age familiar and unfamiliar juvenile mites or of age-synchronized familiar or unfamiliar juvenile mites or of pairs of familiar or unfamiliar larvae. Overall, familiar mites preferentially grouped together and foraged more efficiently, i.e. needed less prey at similar developmental speed and body size at maturity, than unfamiliar mites. Preferential association of familiar mites was also apparent in the inter-exuviae distances. Social familiarity was established by imprinting in the larval stage, was not cancelled or overridden by later conspecific contacts and persisted into adulthood. Life stage had an effect on grouping with larvae being closer together than nymphal stages. Ultimately, optimized foraging during the developmental phase may relax within-group competition, enhance current and future food supply needed for optimal development and optimize patch exploitation and leaving under limited food.

## Introduction

In virtually every organism, environmental constraints experienced during early life have significant effects on phenotypic development and key life history traits such as growth, development, survival, body size or reproduction (for review, see Stearns 1992; Nylin and Gotthard 1998; Metcalfe and Monaghan 2001; Monaghan 2008). For animals, well-documented major contexts include food shortage (e.g., Koedijk et al. 2010; Walzer and Schausberger 2011), adverse abiotic conditions such as extreme temperature and humidity (Van der Linde and Sevenster 2006; Indermaur et al. 2010), predation risk (Indermaur et al. 2010) or an unfavourable social environment (e.g., Beletsky and Orians 1989; Höjesjö et al. 1998). In group-living species, an important stressor during the juvenile developmental phase is the presence of and social interactions with conspecific individuals. This is especially true for species inhabiting small, ephemeral food patches such as tadpoles, nesting species such a birds or predatory mites exploiting patchily distributed prey (Schausberger 2003; Alcock 2005; Danchin et al. 2008; Hawley 2009). Within such patches, conspecific individuals may be competitors for food, space and future mates or may even be mutual predators. Social familiarity among group members is known to alleviate intraspecific conflicts (Utne-Palm and Hart 2000; Delbarco-Trillo et al. 2009). Consequently, many group-living species are able to discriminate familiar and unfamiliar individuals based on prior association (e.g., Waldman 1988; Mateo 2004) and familiar individuals preferentially associate with each other (Chivers et al. 1995). This in turn may affect foraging (Höjesjö et al. 1998), antipredation (Griffiths et al. 2004) and competitive behaviours (Komdeur et al. 2004; Palphramand and White 2007).

At the cognitive level, limited attention theory (Dukas 2002) provides a plausible explanation for preferential association of familiar individuals. Limited attention theory postulates that focusing on a given task has cognitive and associated physiological and behavioural costs with respect to the attention paid to other tasks. Familiar group members require less attention than unfamiliar ones and assorting with familiar individuals should thus lead to increased efficiency in other tasks (Griffiths et al. 2004). Experimental investigations linking the cognitive, behavioural and life history effects of social familiarity during the juvenile phase are lacking.

Here, we hypothesized that a familiar social environment during the juvenile phase positively affects the developmental performance of the group-living, plant-inhabiting predatory mite *Phytoseiulus persimilis* Athias-Henriot. Social familiarity should reduce the cognitive—due to limited attention—and associated physiological and behavioural—due to decreased stress—costs of group living and consequently provide more optimal conditions during this critical phase. *P. persimilis* is a specialized predator of spider mites of the family Tetranychidae, producing dense webs such as the two-spotted spider mite *Tetranychus urticae* Koch*.* Group living is brought about by the predators foraging, reproducing and developing in the spider mite webs (Sabelis 1985; Schausberger and Croft 1999) and mutual attraction (Zhang and Sanderson 1992; authors unpublished). If spider mite availability allows, groups of *P. persimilis* may consist of all life stages (egg, larva, protonymph, deutonymph and adult). The protonymph is the first feeding stage (Schausberger and Croft 1999). Regarding familiarization, contact early in life, in the larval and/or early protonymphal stage (for within-generation familiarization; Schausberger 2004, 2007; authors unpublished), and briefly after oviposition (for mother–offspring familiarization; Schausberger and Croft 2001; Schausberger 2005) is crucial. We predicted that, within groups, familiar juvenile mites should more likely associate with each other than unfamiliar mites. Social familiarity should optimize their foraging behaviour and consequently their developmental performance. In the first experiment, we assessed the influence of familiarity on within-group association behaviour within mixed-age groups (larvae and protonymphs) of *P. persimilis* by determining the familiarity status of each individual’s neighbours and the inter-individual distances of familiar and unfamiliar individuals. In the second experiment, we examined the foraging behaviour and development—from the larval stage until adulthood—of *P. persimilis* living in age-synchronized groups consisting of either familiar or unfamiliar individuals. The larva is the first developmental stage and most sensitive to environmental factors. Hence, to further pinpoint the timing of familiarization, in the third experiment, we assessed the effects of familiarity on general activity and association behaviour of *P. persimilis* larvae.

## Materials and methods

### Origin and rearing of experimental animals

Experimental animals were offspring from females withdrawn from a laboratory-reared population of *P. persimilis* , originally founded with specimens field-collected in Greece. The predatory mites were fed *T. urticae* of mixed life stages, reared on whole bean plants *Phaseolus vulgaris* L., by adding detached spider mite-infested leaves onto rearing units in 3- to 4-day intervals (Vanas et al. 2006). Rearing and experimental arenas were stored at 25 ± 1°C, 60 ± 5% relative humidity and 16:8 h light/dark.

### Arenas and cages

Arenas used to obtain similarly aged predator eggs (henceforth termed oviposition arenas), to generate familiar individuals in experiment 1 (henceforth termed familiarization arenas), and to assess within-group association, foraging and development in experiments 1 and 2 (henceforth termed experimental arenas) consisted of single bean leaves placed adaxial surface down on a water-saturated, filter paper-covered foam cube (5 × 5 × 4 cm) in a plastic box (10 × 10 × 5 cm) half-filled with water (Schausberger and Croft 2001). Strips of moist tissue paper were folded over the edges of the leaves to prevent the mites from escaping. Before adding the predators, each arena was infested with eight *T. urticae* females for 2 days to deposit eggs to be used as prey by the predators. In experiments 2 and 3, individuals were familiarized in artificial cages (henceforth termed familiarization cage), each consisting of a circular cavity (1.5 cm in diameter) drilled in an acrylic plate (0.3 cm in thickness). The cages were covered by gauze at the lower side and on the upper side by a microscope slide held in place by a metal clamp (Schausberger 1997). The same type of cages was used to store the predator eggs before being used in experiment 1. Experiment 3 took place in artificial cages (henceforth termed experimental cages), the basic construction of which was similar to the familiarization cages but consisting of two circular cavities (each 1.5 cm in diameter) spaced 3 cm apart and connected by a 3-mm-wide corridor.

### Influence of familiarity on within-group association behaviour of mixed-age juvenile individuals (experiment 1)

Experiment 1 aimed at assessing the influence of familiarity and life stage (larvae and protonymphs) on within-group association behaviour of mixed (larvae and protonymphs) juvenile life stages of *P. persimilis* held in groups consisting of both familiar and unfamiliar individuals. Each group consisted of two trios, each consisting of two protonymphs and one larva. Individuals of a trio were familiar with each other but unfamiliar to all individuals of the other trio. To generate familiar larvae and protonymphs, 50 to 80 gravid *P. persimilis* females were allowed to oviposit for 4 to 5 h on oviposition arenas. Eggs were collected and transferred to artificial cages. One third of the eggs (prospective larvae in the experiment) was stored in a refrigerator at 8 ± 1°C and 60 ± 5% RH to halt development; two thirds of the eggs (prospective protonymphs in the experiment) were stored in an environmental chamber at 25 ± 1°C and 60 ± 5% RH. After 12 h, six eggs (four prospective protonymphs and two prospective larvae) were placed onto a familiarization arena infested with *T. urticae* eggs, where the larvae hatched and the protonymphs moulted, respectively, after further 24 to 29 h (at 25 ± 1°C, 60 ± 5% RH). Each familiarization arena had an accessible size of ~1 × 1 cm to ensure physical contact among individuals to become familiar.

After another 4 to 6 h, familiar and unfamiliar individuals were marked with different water colour dots on their dorsal shields and the experiment started by placing two trios on an experimental arena. Each experimental arena had an accessible size of ~3 × 3 cm. The position of each individual was recorded after 0.08, 0.5, 2, 4, 6, 8 and 10 h. Recordings were ceased when a larva had moulted to a protonymph. The positions were marked on leaf-shaped sketches drawn on paper and the developmental and familiarity status of the neighbours of each individual and their inter-individual distances were determined at each observation point. The experiment was replicated 20 times.

### Influence of familiarity on foraging and development of age-synchronized juvenile individuals (experiment 2)

In experiment 2, we assessed the effects of familiarity on within-group association behaviour, predation and development of age-synchronized juvenile *P. persimilis* held in groups consisting of either familiar or unfamiliar individuals. A total of 70 to 90 gravid *P. persimilis* females were randomly chosen from the rearing unit, placed on oviposition arenas and allowed to oviposit for 4 h to obtain similarly aged eggs. Six eggs were placed together in a familiarization cage, where the larvae were allowed to familiarize for 7 to 9 h after hatching.

To start the experiment, either three familiar or three unfamiliar larvae were placed together onto an experimental leaf arena. Larvae of familiar trios were taken out of one and the same familiarization cage, whereas each larva of an unfamiliar trio was taken out of a different familiarization cage. Each experimental arena had an accessible area of 2.5 × 2.5 cm and was infested with 50 eggs of *T. urticae*, warranting surplus prey for complete development of each predator of the trio (Vanas et al. 2006). At 25°C, the developmental time of larvae is ~12 to 14 h and that of protonymphs and deutonymphs is ~24 to 30 h each (Schausberger and Croft 1999; Vanas et al. 2006). The arenas were observed hourly around the expected moulting times until all individuals had moulted to the next developmental stage. The number of spider mite eggs eaten and the inter-exuviae distances were recorded as soon as all protonymphs had moulted to deutonymphs and all deutonymphs had moulted to adults, respectively. All exuviae were removed after each moulting bout. The inter-individual distances were measured ~3 h after all larvae had moulted to protonymphs, all protonymphs had moulted to deutonymphs and all deutonymphs had moulted to adults. Familiar and unfamiliar groups were replicated 28 and 26 times, respectively. After all individuals of an arena had reached adulthood, they were mounted on slides and their dorsal shield length, which is an indicator of their body size (Walzer and Schausberger 2011), was measured at 100 × magnification (18 to 24 replicates per sex and familiarity status).

### Association behaviour and development of larvae (experiment 3)

In experiment 3, we assessed the association behaviour of familiar and unfamiliar pairs of *P. persimilis* larvae. To obtain similarly aged *P. persimilis* eggs, three gravid females were randomly chosen from the rearing unit and placed on oviposition arenas for 4 h. Eggs were collected and four eggs were placed together in a familiarization cage, where the larvae hatched after ~48 h. Larvae were allowed to familiarize for 4 to 6 h after hatching.

To start the experiment, two familiar or two unfamiliar larvae were placed into an experimental cage. To remove any loose structures such as dust fibres or chemical contaminants possibly influencing larval behaviours, each cage was cleaned with 75 % ethanol before experiments. The positions of the two larvae and their exuviae and general activity (walking or resting) were recorded after 0.08, 0.5, 2, 4, 6 and 8 h. Assigning the position of the larvae to pre-defined sectors of the cage allowed to precisely estimate the inter-larvae and inter-exuviae distances after the experiment. Familiar and unfamiliar pairs of larvae were replicated 39 and 40 times, respectively.

### Statistical analyses

All statistical analyses were performed using SPSS 15.0.1 for Windows (SPSS Inc., Chicago, IL, USA, 2006). For analyses of experiment 1, we used only replicates where all six individuals remained on the arena throughout the whole experimental period. We used separate G-tests of goodness of fit (Sokal and Rohlf 1995) to compare the observed numbers of first neighbours (i.e. the closest neighbours) being familiar with the predicted numbers (40 % familiar) at each observation point. To compare the likelihood of being familiar among neighbours 1 (i.e. the closest) through 5 (i.e. the fifth closest) over time, we used generalized estimating equations (GEE; binomial distribution, identity link, autocorrelation structure between observation points) and post-hoc pairwise neighbour comparisons of expected marginal means with Šidák correction for multiple comparisons. Similarly, we used GEE (normal distribution, identity link, autocorrelation structure between observation points) to analyse the effects of familiarity and life stage (used as between-subject variables) and time (used as within-subject variable) on the inter-individual distances (post-hoc pairwise life stage comparisons of expected marginal means by least significant difference tests, LSD). Inter-individual distances of unfamiliar and familiar mites were averaged per arena and observation point before analysis.

In experiment 2, we used separate generalized linear models (GLM; normal distribution, identity link) to analyse the effects of familiarity on life stage-specific and total developmental time, sex ratio and dorsal shield length. Sex ratio was expressed as the proportion of females among individuals of an arena and square root-transformed before analysis. Separate GEEs (normal distribution, identity link, autocorrelation between moulting bouts) were used to analyse the effects of familiarity and life stage on predation rate (per hour), inter-individual distances and inter-exuviae distances over time (post-hoc pairwise life stage comparisons of expected marginal means by LSD).

In experiment 3, we used GEE (normal distribution except for activity levels, which had a binomial distribution, identity link, autocorrelation structure between observation points) to analyse the effects of familiarity on inter-larvae distances and activity levels of larvae over time. Activity levels were quantified as the number of moving larvae per cage and observation point. The influence of familiarity on the inter-exuviae distances and moulting times were compared using GLM (normal distribution, identity link).

## Results

### Influence of familiarity on within-group association behaviour of mixed-age juvenile individuals (experiment 1)

**Fig. 1:**
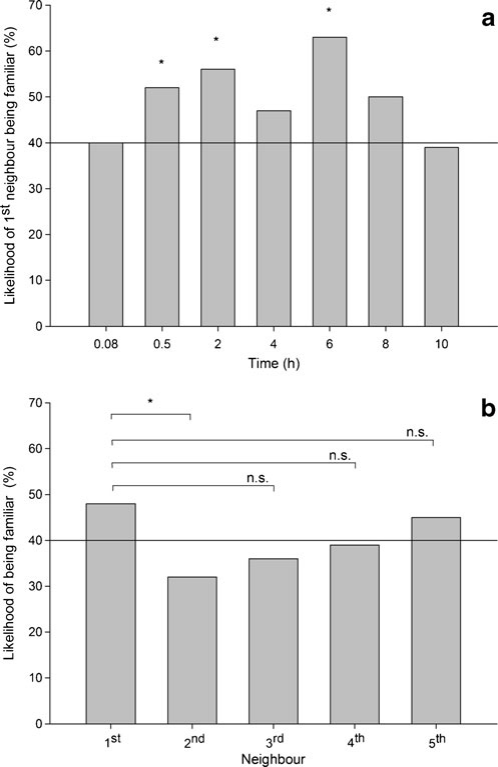
The percentage of first neighbours being familiar over time (**a**) and of first to fifth neighbours being familiar across time (**b**) within groups consisting of both familiar and unfamiliar mixed-age juvenile life stages (larvae and protonymphs) of *P. persimilis*. The reference line represents the expected likelihood of 40 % being familiar. Significance levels (*P < 0.05, n.s. = non-significant) refer to G-tests for goodness of fit in **a** and pairwise Šidák comparisons following GEE in **b**

**Fig. 2:**
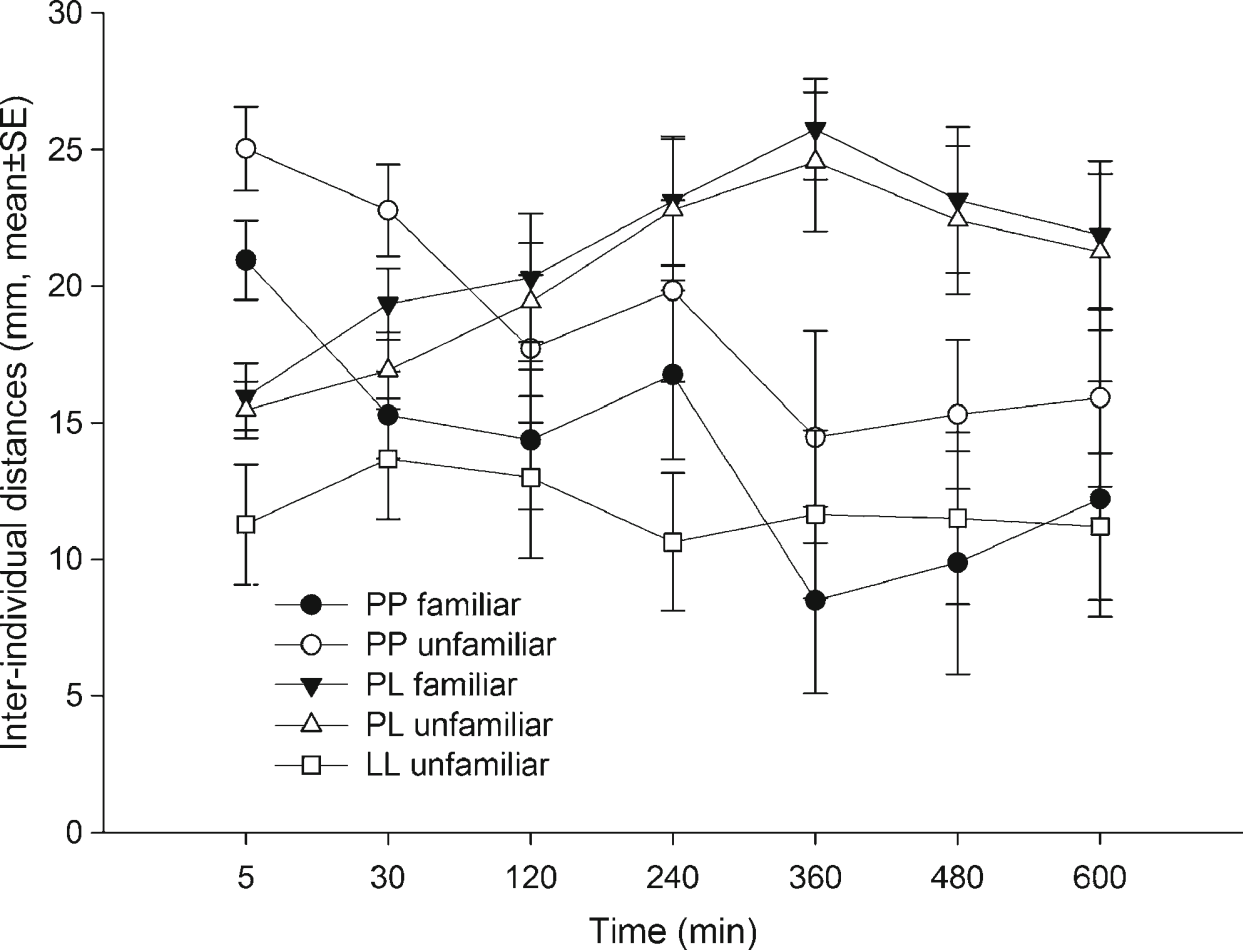
Inter-individual distances within groups consisting of both familiar and unfamiliar mixed-age juvenile life stages (larvae and protonymphs) of *P. persimilis* over time. *LL* larva–larva distances, *PL* protonymph–larva distances and *PP* protonymph–protonymph distances

The likelihood of two neighbouring predatory mites being familiar was significantly higher than expected (predicted 40 % familiar) at three of seven observation points (G-tests for goodness of fit; *P* < 0.05; Fig. 1a). The likelihood of being familiar differed significantly among neighbours (GEE; neighbour: Wald *χ*² = 21.157, *P* = 0.009) and over time (neighbour × time: Wald *χ*² = 3741.859, *P* < 0.001; data not shown here). First neighbours were more likely familiar to the target individual than second neighbours (Šidák, *P* = 0.04), but not third, fourth or fifth neighbours (*P* > 0.05 for each) (Fig. 1b). The likelihood of first neighbours being familiar increased until 6 h and decreased thereafter, while the likelihood of second neighbours being familiar continuously decreased over time. The likelihood of third, fourth and fifth neighbours being familiar did not change over time. The inter-individual distances of familiar and unfamiliar predatory mites pooled across time did not differ but varied differently over time (Table 1; Fig. 2). The distances between familiar juveniles fluctuated more within the first 240 min than those between unfamiliar juveniles. Life stage had a significant effect on the inter-individual distances (Table 1; Fig. 2). Inter-larvae distances (mm, pooled mean ± SE; 13.0 ± 2.03) were lower than the distances between protonymphs (17.32 ± 0.87; LSD, *P* = 0.05) and the distances between protonymphs and larvae (19.65 ± 0.91; LSD, *P* = 0.003). The latter two differed marginally significantly (LSD, *P* = 0.065). During the first 360 min, the inter-protonymphae distances decreased, while the distances between protonymphs and larvae increased. The inter-larvae distances did not change over time (Table 1; Fig. 2).

**Table 1:** Results of generalized estimating equations (GEE) for the effect of social familiarity on inter-individual distances of juvenile *P. persimilis* within groups consisting of both familiar and unfamiliar mixed-age juvenile life stages (larvae and protonymphs) (experiment 1)

Source of variation	Wald *χ*²	df	*P*
Familiarity	4.914	1	0.303
Life stage	9.315	2	<0.010
Familiarity × life stage	0.024	1	0.877
Time × familiarity	220.381	12	<0.001
Time × life stage	59.975	18	<0.001

### Influence of familiarity on foraging and development of age-synchronized juvenile individuals (experiment 2)

**Fig. 3:**
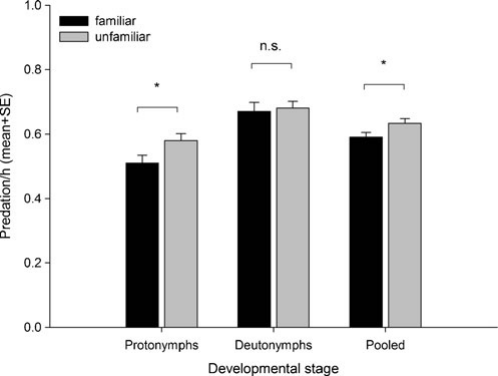
Predation rate (spider mite eggs eaten per hour) of protonymphs and deutonymphs of *P. persimilis* held in age-synchronized groups of either familiar or unfamiliar individuals. Significance levels (*P < 0.05; *n.s.* non-significant) refer to LSD tests following GEE (stage-specific comparisons) and the main effect in GEE (for pooled data)

**Fig. 4:**
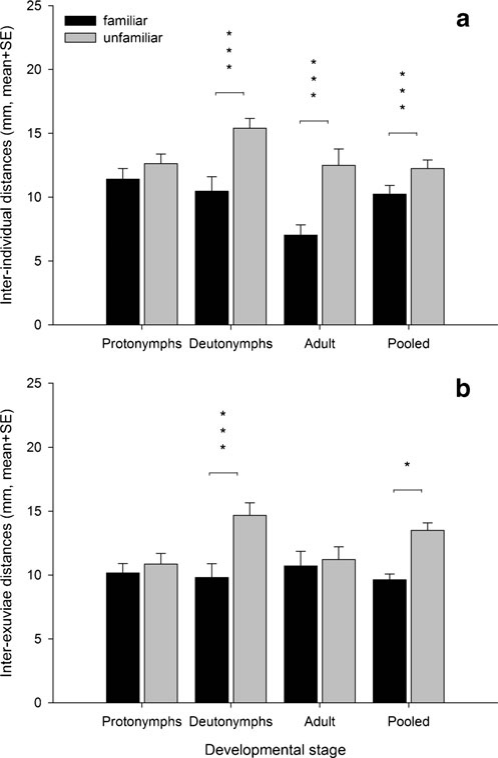
Inter-individual (**a**) and inter-exuviae distances (**b**) during the developmental phase of *P. persimilis* held in age-synchronized groups consisting of either familiar or unfamiliar individuals. Significance levels (*P < 0.05, ***P < 0.001) refer to LSD tests following GEE (stage-specific comparisons) and the main effect in GEE (for pooled data)

The life stage-specific developmental times (eggs + larvae, protonymphs, deutonymphs) did not differ between familiar (h, mean ± SE; 62.08 ± 0.34, 23.54 ± 0.20, 25.44 ± 0.40) and unfamiliar (62.07 ± 0.37, 23.39 ± 0.23, 25.69 ± 0.26) mites (GLM, Wald *χ*_1_² = 1.118, *P* = 0.290). Similarly, the sex ratio of mites held in familiar (female proportion; mean ± SE, 0.62 ± 0.02) and unfamiliar (0.54 ± 0.11) groups (Wald *χ*_1_² = 0.447, *P* = 0.504) and their dorsal shield lengths (μm, mean ± SE; females: familiar 338 ± 42, unfamiliar 341 ± 40, Wald *χ*_1_² = 0.417, *P* = 0.519; males: familiar 286 ± 29, unfamiliar 288 ± 29, Wald *χ*_1_² = 0.581, *P* = 0.446) did not differ. The hourly predation rate was significantly lower in familiar than unfamiliar groups, which was mainly due to differing predation by protonymphs (Table 2; Fig. 3). The inter-individual and inter-exuviae distances were significantly lower in familiar than unfamiliar groups (Table 2; Fig. 4a, b). The significant interaction terms between life stage and familiarity indicate that this was mainly due to inter-individual differences of deutonymphs and adults and inter-exuviae distances of protonymphs, respectively (Table 2; Fig. 4a, b). Life stage had a significant effect on the inter-individual distances (Table 2). The inter-individual distances of adults (mm, mean ± SE 29.26 ± 2.27) were significantly lower than those of protonymphs (36.03 ± 1.70, LSD: *P* = 0.02) and deutonymphs (38.79 ± 2.01, LSD: *P* = 0.03). The inter-individual distances of protonymphs and deutonymphs did not differ (LSD, *P* = 0.32). Life stage had no effect on the inter-exuviae distances (Table 2).

**Table 2:** Results of generalized estimating equations (GEE) for the effects of social familiarity and life stage on predation rate, and inter-individual and inter-exuviae distances of age-synchronized juvenile *P. persimilis* held in groups of either familiar or unfamiliar individuals (experiment 2)

Source of variation	Wald *χ*²	df	*P*
Predation rate			
Familiarity	4.081	1	0.043
Life stage	24.498	1	<0.001
Familiarity × life stage	1.588	1	0.208
Inter-individual distances			
Familiarity	27.682	1	<0.001
Life stage	9.603	2	0.008
Familiarity × life stage	6.857	2	0.032
Inter-exuviae distances			
Familiarity	4.477	1	0.034
Life stage	4.386	2	0.112
Familiarity × life stage	7.865	2	0.020

### Association behaviour and development of larvae (experiment 3)

**Fig. 5:**
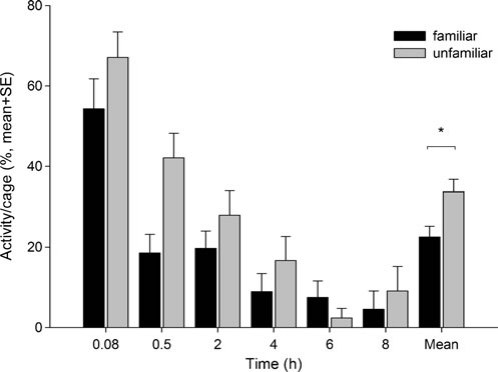
Activity (% larvae moving) within pairs of either familiar or unfamiliar *P. persimilis* larvae over time. Significance level (*P < 0.05) refers to GEE

Familiarity did not have a main effect on the inter-larvae (mm, mean ± SE; 13.61 ± 0.78 for familiar, 15.00 ± 0.86 for unfamiliar; Wald *χ*_1_² = 1.218, *P* = 0.270) and inter-exuviae distances (mm, mean ± SE; 15.12 ± 1.87 for familiar, 18.50 ± 2.02 for unfamiliar; Wald *χ*_1_² = 1.508, *P* = 0.322). However, the distances between unfamiliar larvae increased over time while the distances between familiar larvae remained at about the same level (time × familiarity: Wald *χ*_10_² = 56.748, *P* < 0.001; data not shown here). Unfamiliar larvae were generally more active (Wald *χ*_1_² = 5.601, *P* = 0.018) and stayed active longer (time × activity: Wald *χ*_10_² = 155.176, *P* < 0.001) than familiar larvae (Fig. 5). Familiarity did not affect the developmental times (min, mean ± SE; 426.00 ± 20.05 for familiar and 423.96 ± 23.47 for unfamiliar, Wald *χ*_1_² = 0.004, *P* = 0.947).

## Discussion

Familiarity had significant effects on within-group association behaviour and foraging traits of juvenile *P. persimilis*. In mixed-age groups of familiar and unfamiliar individuals, familiar individuals preferentially associated with each other. Life stage influenced the association behaviour, with larvae being much closer together than protonymphs to each other or protonymphs to larvae. In age-synchronized groups, the inter-individual and inter-exuviae distances were lower within groups of familiar mites than within groups of unfamiliar mites. At similar developmental speed and body size at maturity, juvenile mites held in familiar groups foraged more optimally, i.e. needed less prey items during the developmental phase, than juvenile mites held in unfamiliar groups.

The ability of *P. persimilis* to social imprinting (Mateo 2004) and discriminate familiar from unfamiliar conspecifics has been observed in various contexts such as kin cannibalism (Schausberger and Croft 2001; Schausberger 2004, 2007), egg placement (Schausberger 2005, 2007) and group living of adult females (authors unpublished). Nevertheless, it was unclear how long memory of familiar individuals persists after familiarization early in life and whether familiarity may be flexibly updated later in life. The setups of experiments 2 and 3 allow to pinpoint the timing of imprinting to the larval stage (Schausberger 2007). The consistent behavioural changes induced by larval familiarization point at the existence of a sensitive familiarization period in the larval stage and memory persisting into the adult stage. Post-larval conspecific encounters did not override larval familiarization. In experiment 3, behavioural differences between familiar and unfamiliar larvae were already apparent after a few hours of familiarization in familiarity-dependent activity levels and inter-larvae distances over time. Lower activity of familiar than unfamiliar larvae is similar to related findings for adult females (authors unpublished) and may be interpreted as an indicator of a less stressful social environment. Lowered activity may allow to shift attention to anti-predator vigilance as has been shown for familiar trout (Griffiths et al. 2004) or may reduce the likelihood of encountering possible predators. In experiment 2, moulting did not cancel the effects of familiarity established in the larval stage. In contrast, these effects were getting more pronounced through development, suggesting that post-larval re-familiarization either did not occur or, if it occurred, did not override larval familiarization.

The effects of familiarity were less pronounced in larvae (experiment 3) than in the nymphal stages observed in experiments 1 and 2, probably for two main reasons. In experiment 3, larvae could only familiarize for a few hours before the experiment took place, whereas they could familiarize throughout the larval stage in experiments 1 and 2. The larva is the first developmental stage, non-feeding and quite vulnerable to intraguild predation and cannibalism (Schausberger 2003). Larvae generally tend to stay close together and remain rather immobile until moulting to the more mobile, feeding and thus more defensive protonymph, probably to avoid predator encounters and benefit from the dilution effect (e.g., Foster and Treherne 1981). Co-occurring protonymphs are possible predators of larvae, which may explain why in mixed-age groups the distances between larvae and protonymphs were the largest (experiment 1). It is thus likely that, in experiment 1, the propensity of larvae to associate with a life stage companion somewhat diminished the effects of familiarity or made these effects more subtle and difficult to measure.

We hypothesized that social familiarity reduces the interrelated cognitive, physiological and behavioural costs of group living and should thus lead to optimization of juvenile foraging and development. The developmental times of individuals held in familiar groups were not different from individuals held in unfamiliar groups, but familiar mites, in particular protonymphs, had lower predation rates than unfamiliar ones. Familiar mites thus foraged more optimally than unfamiliar mites because they needed less prey items to complete development at a similar speed and body size at maturity. Optimal foraging theories predict that selection favours foraging strategies, which optimize energy gain by maximizing food intake rate and/or minimizing handling and/or searching times (Stephens and Krebs 1986; Giraldeau 2008). For example, Griffiths et al. (2004) observed that socially familiar fish consumed more food items per time unit than unfamiliar fish and suggested that familiarity allowed to switch attention from within-group aggression to foraging. However, higher feeding rates do not necessarily indicate enhanced foraging efficiency and do not necessarily translate into enhanced life history traits (Stearns 2000; Illius et al. 2002; Lawson-Balagbo et al. 2008). In our study, familiar mites apparently optimized energy gain per prey item eaten or reduced energy invested in other activities, allowing to feed less at the same developmental speed. Social familiarity did not shorten the developmental times, probably indicating that the developmental times were at their optimum. Due to trade-offs between optimal tissue formation and rapid development, life history theories commonly predict that the optimal developmental time is usually not the fastest possible (e.g., Zwaan et al. 1995; Metcalfe and Monaghan 2001; Šešlija and Tucić 2003; Walzer and Schausberger 2011).

Proximate reasons of social familiarity amending foraging behaviour include reduced energy needed for neighbour assessment, amended prey handling or more thorough sucking out of prey due to reduced neighbour disturbance. Possible ultimate benefits arising from feeding less at optimal developmental speed are manifold. A lower feeding rate may lead to a longer lasting prey patch and therefore provide food for longer time or for more individuals. Suboptimal exploitation of a finite food source could cause individuals to disperse prematurely or in a life stage or phase that is less well adapted to disperse and find a new prey patch. Lower feeding rates may not only benefit current residents of a prey patch but may also warrant future food supply for offspring. *P. persimilis* females are known to adjust oviposition and prey patch leaving to optimize prey availability for their offspring (Vanas et al. 2006). Furthermore, if familiarity is used as a proxy of kin (Waldman 1988; Mateo 2004; Schausberger 2007), optimized foraging behaviour may result in more food items left for collateral kin and may thus increase indirect fitness (Hamilton 1964). Enhanced prey utilization may not only optimize food availability during juvenile development but also influence subsequent life history trajectories and performance after reaching adulthood and induce trans-generational effects (Beckerman et al. 2002; Plaistow et al. 2006).
